# Improving nurses’ knowledge of managing endotracheal tube cuff pressure in intensive care units: A quasi-experimental study

**DOI:** 10.4102/hsag.v25i0.1479

**Published:** 2020-12-18

**Authors:** Ferestas Mpasa, Dalena R.M. van Rooyen, Danie Venter, Portia Jordan, Wilma ten Ham-Baloyi

**Affiliations:** 1 Department of Nursing Science, Faculty of Health Sciences, Nelson Mandela University, Port Elizabeth, South Africa; 2 Department of Nursing and Midwifery Science, Mzuzu University, Luwinga, Malawi; 3 Faculty of Health Sciences, Nelson Mandela University, Port Elizabeth, South Africa; 4 Department of Nursing and Midwifery, Faculty of Medicine and Health Sciences, Stellenbosch University, Cape Town, South Africa

**Keywords:** cuff pressure management, evidence-based practice, intensive care units, knowledge, nursing

## Abstract

**Background:**

Previous studies conducted on nurses’ knowledge regarding endotracheal tube cuff pressure revealed that there were differences in intensive care nurses’ knowledge, leading to varying practices.

**Aim:**

This study aimed to evaluate how an educational intervention based on the existing evidence-based guidelines, using both passive and active implementation strategies, could improve the knowledge of nurses regarding managing endotracheal tube cuff pressures in Malawian intensive care units.

**Setting:**

Six functional ICUs (four public and two private) in Malawi.

**Methods:**

The study followed a quasi-experimental, pre- and post-test design using an educational intervention. Intensive care nurses of six functional intensive care units in Malawi were randomly assigned to two intervention groups. Both groups received a half-day educational session, a printed version of the evidence-based guidelines, a printed and laminated summary of the guidelines and a related algorithm. Additionally, Intervention 2 group received four monitoring visits. Pre- and post-test questionnaires were conducted between February and August 2016. Descriptive and inferential data analyses (a chi-square test and *t*-test) were utilised.

**Results:**

An improvement in knowledge was observed on the nursing care practices for the management of endotracheal tube cuff pressure for both groups following the educational intervention, although only the results comparing Intervention 2 group participants indicate that the level of knowledge was significant (*t*[df = 48] = 2.08, *p* = 0.043, *d* = 0.59).

**Conclusion:**

Implementation of a formal training and mentorship programme for Malawian intensive care nurses would be of great benefit to enhance the knowledge and skills managing endotracheal tube cuff pressure. Follow-up studies would also assist in understanding how guidelines could be implemented most effectively to achieve better knowledge outcomes.

## Introduction

Mechanical ventilation, which is used to treat life-threatening conditions, has become a way of managing critically ill patients (Zamzam et al. 2015). However, regardless of the advancement modalities of mechanical ventilation, the technology may contribute to physiological and psychological complications if not well-managed (Dellaca, Veneroni & Farre 2017). Complications such as aspiration pneumonia, tracheal stenosis, adhesions and tracheal malacia, general discomfort and sleep disturbance have been reported (Feng, Ye & Doyle 2015; Goligher et al. 2018; Higgs et al. 2018; Pham, Brochard & Slutsky 2017). The complications can be minimised or prevented if evidence-based guidelines are used in the management of endotracheal tube (ETT) cuff pressure (Higgs et al. 2018). The management of ETT cuff pressure in mechanically ventilated adult patients requires nurses working in intensive care units (ICUs) to have the responsibility of using evidence-based guidelines that direct them in keeping ETT cuff pressure within the normal ranges of 20 cmH_2_O – 30 cmH_2_O or 18 mmHg – 22 mmHg (Sanaie et al. 2019). Unlike in many other, predominantly resource-rich countries with sufficient staffing, in Malawi, this is performed by ICU nurses. Furthermore, evidence-based guidelines enable the standardisation of care regarding the management of ETT cuff pressure so that neither over- nor under-inflation of the cuff occurs (Sanaie et al. 2019).

Evidence-based guidelines are defined as systematically developed statements based on the best evidence of recommended practice in a specific clinical or health work environment (Registered Nurses Association Ontario 2012). In the ICU, the use of evidence-based guidelines is essential, as they ensure the successful management of ETT cuff pressure and significantly reduce the risk of tracheal injuries in mechanically ventilated patients (National Institute for Health and Care Excellence [NICE] 2019).

To enhance the uptake and implementation of evidence-based guidelines, multiple strategies can be used. These strategies include passive methods, such as printed material and formal lectures, as well as active methods, such as educational sessions, audits, feedback, educational outreach visits, academic detailing and videoconferencing (Villarosa et al. 2019).

Strategies in the form of educational programmes should be implemented to enhance the nurses’ knowledge. This is especially important as previous studies conducted on nurses’ knowledge regarding ETT cuff pressure revealed differences in ICU nurses’ knowledge, leading to varying practices (Abubaker et al. 2019; Jordan 2011; Jordan, Van Rooyen & Venter 2012; Mohammed et al. 2016; Mwakanyanga, Masika & Tarimo 2018). A study by Mohammed et al. (2016) showed that an educational programme including discussion groups and formal lectures accompanied by suitable teaching aids, such as handouts, posters, coloured pictures and a programme booklet, improved the knowledge of the nurses regarding ETT cuff pressure.

### Problem statement

The first author observed that 50% (250) of approximately 500 adult patients admitted annually to six functional ICUs in Malawi were mechanically ventilated. Furthermore, three quarters of these six ICUs did not have evidence-based guidelines accessible to the intensive care nurses, guiding them in the management of ETT cuff pressure in mechanically ventilated adult patients. However, no study regarding the management of ETT cuff pressure in ICUs had been conducted in Malawi. In addition, the first author observed that the non-availability of guidelines for the management of ETT cuff pressure in some ICUs often led to inadequate knowledge of ETT cuff pressure practices. These observations highlighted the need for evaluating how an educational intervention based on the existing evidence-based guidelines, using passive versus active implementation strategies, could improve the knowledge of nurses regarding managing ETT cuff pressures in Malawian ICUs.

### Aim

This study aimed to evaluate how an educational intervention based on the existing evidence-based guidelines, using both passive and active implementation strategies, could improve the knowledge of nurses regarding managing ETT cuff pressures in Malawian ICUs.

### Definition of key concepts

#### Endotracheal tube cuff pressure

Endotracheal tube cuff pressure is the amount of pressure inside the cuff created by inflating it in order to create a seal to the entry of pharyngeal contents into the trachea and enhance positive pressure ventilation (Lizy et al. 2011). In this study, the ETT cuff pressure is the pressure inside the tube cuff ranging between the acceptable limits of 20 cmH_2_O and 30 cmH_2_O as evidenced by the literature.

#### Mechanical ventilation

Mechanical ventilation (invasive and non-invasive) is the primary method of supporting organ function by using an artificial (machine) respirator in patients treated in ICUs. Non-invasive mechanical ventilation is where the ventilator support is provided without the use of an ETT, whereas invasive mechanical ventilation refers to the use of an ETT (Kübler et al. 2013). In the present study, mechanical ventilation will refer to the use of invasive mechanical ventilation using an ETT.

#### Intensive care unit

An ICU is a specialised unit in a hospital where critically ill patients are admitted. The environment of an ICU is highly technological, requiring the nurses to have a broad knowledge base and a high level of decision-making skills as they care for patients and their families who are in vulnerable circumstances (De Beer, Baysewicz & Bengu 2011). In this research study, ICUs will include those in both public and private hospitals in Malawi.

#### Management

Robbins and Coulter (2007) define management as applying activities that relate to obtaining the most outputs from the least inputs to a situation. Management refers to the treatment given to patients and the nursing care rendered. For the purpose of this research study, management of ETT cuff pressures will include the following activities performed by the nurses working in the ICUs: (1) the frequency of monitoring of the ETT cuff pressure, (2) the methods used for monitoring the ETT cuff pressure, (3) management of leaks, (4) how much pressure should be maintained in the ETT cuff, (5) patient’s position when monitoring the ETT cuff pressure and (6) the amount of air required to inflate the ETT cuff pressure.

## Research design and methods

### Design

This study employed a quasi-experimental design, with pre- and post-tests, using an educational intervention with two groups of nurse participants.

### Setting

The study was conducted in the six functional ICUs (four public and two private) in Malawi. The average bed capacity of the hospitals was 4, except for one public ICU that had a bed capacity of 6. In both set-ups, there were more nurses trained on the job than those who had undergone formal intensive care nursing specialisation training.

### Participants

An independent observer divided the four public ICUs and the two private ICUs into two intervention groups so that each group contained one private and two public ICUs, using simple randomisation. Firstly, the names of one private and two public hospitals were randomly picked from two bowls containing the names of private and public hospitals, respectively. The names of the first set of hospitals were then placed in an envelope and the second set of hospitals was put in another envelope. These envelopes were then placed in a basket, and the first envelope chosen by the same independent observer would be the intervention group receiving the full educational intervention, using both active (monitoring visits) and passive implementation strategies.

**FIGURE 1:**
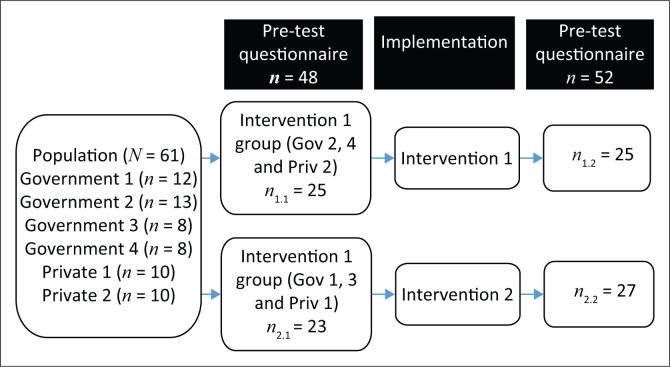
Sampling framework per intervention group.

For both the pre- and post-test questionnaires, the targeted population comprised the nurses working in the ICUs for the duration of the study. The total number of nurses working in the six ICUs during the study was 61. The small population made it unnecessary to calculate a minimum sample size, as the research plan was to include as many population as possible. Because of the small number of nurses available in Malawi, convenience sampling was used to include as many as nurses as possible during the study. In total, 48 nurses were included in the pre-test questionnaire and 52 participants were included in the post-test questionnaire (see Figure 1).

### The intervention

An educational intervention was developed based on the existing evidence-based guidelines for the management of ETT cuff pressure in mechanically ventilated adult patients (Jordan 2011). These guidelines were selected as they were developed in the context of a low- and middle-income country, considering the resource constraints experienced in this context. As the use of multifaceted; strategies, and particularly including active strategies, has proven to enhance staff compliance with evidence-based interventions in the ICU, and subsequently improved knowledge and practices in this context (Hawe et al. 2009; Villarosa et al. 2019), an educational intervention was developed including multiple implementation strategies with both passive and active strategies. The educational intervention consisted of a half-day educational session using a printed version of the evidence-based guidelines, a summary of these guidelines, a related algorithm, a PowerPoint presentation explaining the concepts of the guidelines and four monitoring visits. These guidelines were reviewed and validated for their relevance to the Malawian ICU context by six professional experts who were purposively selected, based on their ICU experience in Malawi, using the Appraisal of Guidelines for Research & Evaluation (AGREE) II tool (The AGREE Research Trust 2013). The experts comprised one lecturer teaching critical care nursing at a university level, one anaesthetist in a central hospital, one ICU nursing unit manager (ICU trained) and three nurses working in three different ICUs.

A PowerPoint presentation was developed that was used for a half-day orientation to the concepts of the evidence-based guidelines. The PowerPoint presentation included a definition of mechanical ventilation, indications for mechanical ventilation, complications of mechanical ventilation, the management of ETT cuff pressure, the complications of ETT cuff pressure and the prevention of ETT cuff pressure complications. A summary of the evidence-based guidelines and an algorithm for the management of ETT cuff pressure in mechanically ventilated adult patients were printed and laminated. The monitoring visits involved one-on-one sessions between the first author and the ICU nurses regarding the importance of adhering to the evidence-based guidelines, how the nurses were coping with the implementation of the guidelines and the problems faced in implementation of these. Any uncertainties linked to the guidelines were clarified during these sessions. The educational intervention was reviewed regarding its relevance by the second author who is an experienced ICU nurse.

Intervention 1 group (which served as the control group) received only a half-day educational session using printed and laminated evidence-based guidelines, a summary of these, a related algorithm, a PowerPoint presentation and no monitoring visits (only passive implementation strategies). Intervention 2 group received the full educational intervention using both active (monitoring visits) and passive implementation strategies.

### Data collection instrument

For the pre- and post-test questionnaires, structured questionnaires were used, adapted with permission from Jordan (2011) by the first author. Both pre- and post-test questionnaires had two sections. Section A (five items) involved the demographic data of the nurses working in the ICUs (including gender, age, specialisation in ICU, type of hospital and number of years working in the ICU). Section B (six items) captured knowledge related to the nursing care practice for the management of ETT cuff pressure and methods used as well as complications of under- and overinflation of the ETT cuff (two items). The items included in the questionnaires were as follows: (1) frequency of monitoring ETT cuff pressure, (2) methods used to monitor ETT cuff pressure, (3) position of the patient during monitoring of ETT cuff pressure, (4) recommended ETT cuff pressure to be maintained, (5) management of audible leaks, (6) amount of air used to inflate an ETT cuff, (7) complications of under-inflation of the ETT cuff pressure and (8) complications of overinflation. An overall knowledge score was calculated for each respondent, based on the percentage of correct responses to the aforementioned items. The correct answer for each item was decided based on the existing evidence-based guidelines for the management of ETT cuff pressure in mechanically ventilated adult patients in agreement with all authors.

### Data collection

Data from the pre-test questionnaire were collected in February–March 2016 using a hand-delivered self-administered questionnaire. A detailed explanation was given to each participant regarding the objectives of the study prior to their signing a consent form agreeing to participate. The participants were assured that the study would not pose risks to themselves or to the patients. Participants were requested not to discuss the questions with their colleagues for the answers to truly reflect their knowledge. Questionnaires were collected immediately after completion and secured in an opaque envelope to ensure confidentiality of information. Because of the busy schedules and the nurses’ shifts in the ICUs, the first author had to visit each ICU several times to complete data collection during the nurses’ day shifts. Data from the staff on night shifts were collected just after handover to the day shift at the end of the night shift or just after handover from the day shift at the beginning of the night shift.

After the pre-test questionnaire was completed by both groups (*n*_1.1_ and *n*_2.1_), the educational intervention was implemented. Both groups were given the half-day educational session using a PowerPoint presentation so that both groups would be at the same level of knowledge pertaining to the guideline at the beginning of the implementation of the educational intervention (Handley et al. 2018). In addition, both groups received a printed version of the evidence-based guidelines, and a summary of the evidence-based guidelines and the algorithm were printed in bright colours, laminated and posted in high traffic, easy-to-see areas such as at the foot or head of the patient’s bed, or on ICU doors (passive implementation strategies). Additionally, for Intervention 2 group, four monitoring visits using one-on-one sessions with the ICU nurses (active implementation strategy) were conducted during 3 months of implementation (April 2016 – June 2016). Data for the post-test questionnaire were collected between July and August 2016 for both groups (*n*_1.2_ and *n*_2.2_) using the same data collection procedure as the pre-test questionnaire.

### Data analysis

Data were analysed using descriptive and inferential statistics. The first author was assisted by the third author, a senior statistician consultant, using visual basic applications in Excel. Means, frequencies and standard deviations were used for the descriptive analysis, whilst inferential statistics (such as the χ^2^ and *t* tests) was used to analyse the knowledge questions where the χ^2^ test was based on sample frequencies (two or more per sample) and was conducted as a further investigation of the differences between the two intervention groups, and the *t* test was based on sample mean values (one value per sample) which allowed for looking at the means only instead of distribution of the scores. This facilitated an examination of the level of significance of the nurses’ knowledge improvement, the *t* distribution and degree of freedom and the determination of the probability of difference between the two intervention groups. Significance was at *p* < 0.05.

### Validity, reliability and rigour

The pre- and post-test questionnaires were adapted with permission from Jordan (2011). To establish the robustness of the questions in the questionnaires, a pilot study was conducted with 14 participants (Jordan 2011, Jordan, Van Rooyen & Venter 2012). A Cronbach’s alpha calculation was conducted for the eight items used in the questionnaire, which were not multiple response items but were coded 0 = incorrect and 1 = correct based on participants’ answers. An acceptable Cronbach’s alpha of 0.60 was achieved for these items (Nunnally & Bernstein 1994).

### Ethical consideration

Ethical clearance was obtained from the Faculty of Health Sciences’ Postgraduate Studies Committee (ethics number: H14-HEA-NUR-001) at the Nelson Mandela University and from the National Health Sciences Research Ethics Committee in Malawi (ethics number: 15/3/139). Approval was obtained from each of the six hospitals involved in the study. Consent was requested from all the participants and participation was voluntary. The questionnaires did not include any confidential data.

## Results

Sixty-one questionnaires were delivered, which were completed by 48 participants (for the pre-test questionnaire) and 52 participants (for the post-test questionnaire), amounting to the response rates of 79% and 85%, respectively. The pre-test questionnaire yielded a Cronbach’s alpha of 0.50 for the knowledge score, whilst the post-test questionnaire resulted in a score of 0.47. An acceptable reliability interval score of 0.47, was considered sufficiently close to the required score of 0.50. However, the obtained score was at a lower range of the acceptable reliability interval.

### Demographic characteristics of participants

Most of the nurses who completed both the pre- and post-test questionnaire in both intervention groups were women. Most participants were between 25 and 39 years old. Not even a quarter of the nurses were specialised in intensive care nursing. Most nurses were from public hospitals and almost half of the participants had worked in the ICU for only a period of 1–4 years. Table 1 outlines the demographics of the respondents in both the pre-and post-test questionnaires.

**TABLE 1:** Demographic characteristics of the participants (pre- and post-test questionnaires).

Demographic items	Intervention 1 group (*n*_1_)				Intervention 2 group (*n*_2_)				Total	
	Pre		Post		Pre		Post			
	*n*_1.1_	%	*n*_1.2_	%	*n*_2.1_	%	*n*_2.2_	%	*n*	%
**Gender**										
Male	3	12	8	32	5	22	4	15	-	-
Female	22	88	17	68	18	78	23	85	-	-
**Age in years**										
< 25 years	4	16	0	0	1	4	4	16	9	9
25–29 years	8	32	8	32	5	22	8	32	29	30
30–39 years	9	36	13	52	7	30	6	24	35	36
40–49 years	2	8	3	12	5	22	5	20	15	15
50–59 years	2	8	1	4	4	17	2	8	9	9
60+ years	0	0	0	0	1	4	0	0	1	1
**Specialised in ICU**										
Yes	8	32	3	12	6	26	4	15	21	21
No	17	68	22	88	17	74	23	85	79	79
**Hospital**										
Government 1	-	-	-	-	8	35	13	48	-	-
Government 2	11	44	10	40	-	-	-	-	-	-
Government 3	-	-	-	-	6	26	6	22	-	-
Government 4	7	28	6	24	-	-	-	-	-	-
Private 1	-	-	-	-	9	39	8	30	-	-
Private 2	7	28	9	36	-	-	-	-	-	-
**No. of years working in ICU**										
< 1 year	9	36	5	20	7	30	14	52	35	35
1–4 years	11	44	17	68	9	39	6	22	43	43
5–9 years	3	12	2	8	5	22	3	11	13	13
10–14 years	1	4	1	4	0	0	2	7	4	4
> 14 years	1	4	0	0	2	9	2	7	5	5

ICU, intensive care unit.

### Knowledge of the management of endotracheal tube cuff pressure

Questionnaire responses with respect to the practices related to the management of ETT cuff pressure were statistically treated as knowledge questions and are outlined in Table 2. The results of the post-test questionnaire on the respondents’ knowledge score of nursing care practices for the management of ETT cuff pressure were superior to those of the pre-test questionnaire. An increase was observed in the number of correct answers from Intervention 2 group for all eight of the relevant questionnaire items except for ‘B5 Management of audible leaks’ (where there was a decline from 96% to 68%). The improvement for Intervention 1 group ranged between 6% (35% – 42%) for ‘B7 Complications of under-inflation of the ETT cuff pressure’ and 68% (8% – 76%) for ‘B1 Frequency of monitoring ETT cuff pressure’. The improvement for Intervention 2 group ranged between 10% (36% – 46%) for ‘B7 Complications of under-inflation of the ETT cuff pressure’ and 40% (30% – 70%) for ‘B1 Frequency of monitoring ETT cuff pressure’ (see Table 2).

**TABLE 2:** Responses to questionnaire items relating to knowledge of nursing care practices for the management of endotracheal tube cuff pressure.

Knowledge items	Intervention 1 group (*n*_1_)				Intervention 2 group (*n*_2_)			
	Pre		Post		Pre		Post	
	*n*_1.1_	%	*n*_1.2_	%	*n*_2.1_	%	*n*_2.2_	%
**B1 Frequency of monitoring ETT cuff pressure**								
2 hourly	7	28	0	0	5	22	4	15
4 hourly	2	8	1	4	0	0	0	0
6 hourly	4	16	1	4	0	0	1	4
12 hourly?	2	8	19	76	7	30	19	70
Whenever a leak occurs	8	32	2	8	8	35	2	7
Never	2	8	2	8	3	13	1	4
**B2 Methods used to monitor ETT cuff pressure**								
Listening for air leaks	8	32	2	8	7	30	4	15
Use of minimal occlusive volume technique (MOV)	0	0	2	8	0	0	6	22
Use of minimal leak technique (MLT)	2	8	0	0	0	0	2	7
Use of an aneroid manometer?	0	0	6	24	1	4	8	30
Palpating the cuff with fingers to estimate the pressure in the cuff	15	60	15	60	15	65	7	26
**B3 Position of the patient during the monitoring of ETT cuff pressure**								
Supine and flat	5	20	2	8	7	32	0	0
Supine to up to 30°	7	28	8	33	2	9	5	19
Supine 30°–45°?	7	28	10	42	5	23	16	62
Supine 45°–90°	1	4	0	0	0	0	0	0
Lateral position	1	4	2	8	0	0	0	0
No change in position	4	16	2	8	8	36	5	19
**B4 Recommended ETT cuff pressure to be maintained**								
18–22 cmH_2_O	6	24	15	60	2	9	10	38
23–25 cmH_2_O	3	12	0	0	3	13	2	8
26–30 cmH_2_O?	1	4	7	28	2	9	9	35
> 31 cmH_2_O	0	0	0	0	2	9	0	0
Do not know	15	60	3	12	14	61	5	19
**B5 Management of audible leaks**								
Inflate air despite the air already in the cuff till a leak is not heard any more?	9	36	12	48	6	26	10	40
Notify the physician/anaesthetic doctor/surgeon?	8	32	9	36	16	70	7	28
Change position of ETT and patient	1	4	0	0	0	0	0	0
Assess and palpate for cuff pressure	7	28	4	16	1	4	8	32
**B6 Amount of air used to inflate an ETT cuff**								
2 mL	7	28	5	20	7	30	4	15
5 mL	5	20	2	8	3	13	5	19
10 mL?	3	12	6	24	1	4	7	27
20 mL	1	4	0	0	0	0	0	0
Inflate till leak disappears despite the air volume already present in the cuff	3	12	8	48	8	35	10	38
Do not know	6	24	0	0	4	17	0	0
**B7 Complications of under-inflation of the ETT cuff pressure**								
Aspiration of gastric contents	11	44	8	33	11	48	8	32
Increase chances of ventilator-associated pneumonia	5	20	5	21	4	17	5	20
All the above?	9	36	11	46	8	35	11	41
None of the above	0	0	0	0	0	0	1	4
**B8 Complications of overinflation of the ETT cuff pressure**								
Tracheal erosion	3	12	1	4	9	39	0	0
Tracheal stenosis	5	20	7	28	7	30	12	46
Tracheal rupture	6	24	1	4	2	9	3	12
Tracheal innominate artery fistula	1	4	2	8	0	0	0	0
All the above?	9	36	14	56	5	22	10	38
None of the above	1	4	0	0	0	0	1	4

Note: Correct responses are indicated using a dagger sign.

ETT, endotracheal tube.

Statistics for the knowledge scores calculated for each respondent as the percentage of correct responses to the questionnaire items listed in Table 2 are reported in Tables 3 and 4. As indicated in Table 3, the knowledge scores of the post-test participants were superior to that of the pre-test participants, with an increase of 12% (32% – 44%) for Intervention 1 group and 24% (13% – 37%) for Intervention 2 group in the number of respondents with a knowledge score of 40 out of 100 or better. The improvement was, however, not statistically significant for either group (see Table 3).

**TABLE 3:** Contingency tables – Knowledge scores per group.

Score	Intervention 1 group (*n*_1_)								Intervention 2 group (*n*_2_)							
	Pre				Post				Pre				Post			
	*n*_1.1_	%	*n*_1.1_	%	*n*_1.2_	%	*n*_1.2_	%	*n*_2.1_	%	*n*_2.1_	%	*n*_2.2_	%	*n*_2.2_	%
Very low 0–19	13	52	17	68	10	40	14	56	18	78	20	87	12	44	17	63
Low 20–39	4	16			4	16			2	9			5	19		
Average 40–60	7	28	7	28	11	44	11	44	3	13	3	13	10	37	10	37
High 61–80	1	4	1	4	0	0	0	0	0	0	0	0	0	0	0	0
Very high 81–100	0	0			0	0			0	0			0	0		
**Total**	**25**	**100**	**25**	**100**	**25**	**100**	**25**	**100**	**23**	**100**	**23**	**100**	**27**	**100**	**27**	**100**

Inferential statistics (comparing score categories 0–39 and 40–80); Within intervention 1 group – Pre and post, *χ*^2^(1) = 0.76, *p* = 0.382; Within intervention 2 group – Pre and post, *χ*^2^(1) = 3.72, *p* = 0.054; Between intervention 1 and 2 groups – Pre, *χ*^2^(1) = 2.44, *p* = 0.119; Between intervention 1 and 2 groups – Post, *χ*^2^(1) = 0.26, *p* = 0.609.

**TABLE 4:** *T*-test by pre- and post-test questionnaires for both intervention groups – Knowledge.

Group	Test	*n*	Mean	SD	Difference	*t*	*p* (*df* = 48)	Cohen’s *d*
Intervention 1	Pre	25	25.14	22.69	6.29	1.00	0.323	0.28
	Post	25	31.43	21.82				Small
Intervention 2	Pre	23	16.15	15.11	10.84	2.08	0.043	0.59
	Post	27	26.98	20.71				Medium

M, mean; SD, standard deviation; t, T-test; p, p-value; df, degrees of freedom.

As indicated in Table 4, the mean knowledge score of the post-test participants was 6.29 and 10.84 higher than that of the pre-test participants in Intervention groups 1 and 2, respectively. The improvement was not statistically significant for Intervention 1 group, but it was significant for Intervention 2 group (see Table 4).

## Discussion

Management of ETT cuff pressure is an essential aspect of nursing care of intubated patients, and the importance of complications related to undesirable cuff pressure cannot be overemphasised (Gheshlaghi 2015). Nurses are at a patient’s bedside around the clock, and it is expected from them to be knowledgeable of the complications that can arise when the ETT cuff is under- or overinflated (Jun, Kovner & Stimpfel 2016). Adequate knowledge of practices related to the management of ETT cuff pressure is, therefore, imperative. The findings of this study show that the post-test questionnaire respondents’ knowledge of nursing care practices for the management of ETT cuff pressure was superior in both intervention groups. However, the overall knowledge score of most participants in both groups after the post-test was low, but improved significantly for Intervention 2 group, using both passive and active implementation strategies.

Generally, our study results showed that knowledge regarding specific practices was particularly low. Firstly, fewer nurses had knowledge about the correct range of ETT cuff pressure of 22 mmHg – 30 mmHg in both groups, which improved slightly, but not significantly for both groups. Gilliland, Perrie and Scribante (2015) stress that tracheal perfusion pressure estimated to be 22 mmHg – 30 mmHg should not be exceeded by the ETT cuff pressure. Exceeding tracheal perfusion pressure impedes tracheal mucosa circulation, which leads to ischemia and the development of lesions.

Secondly, fewer participants indicated that they would continue cuff inflation, irrespective of the volume of air inserted, or continue cuff inflation, notifying the physician in the pre-test period as well as the post-test where a reduction in knowledge, although not significant, was shown for Intervention 2 group. Leaks of ETTs in mechanical ventilation cause a loss of volume for positive pressure ventilation and low oxygenation. Sometimes audible leaks are used as a base for monitoring ETT cuff pressure by nurses caring for mechanically ventilated patients (Letvin et al. 2018). Although the comparison of the groups was not statistically significant, our study revealed an improvement in nursing care practice regarding the management of audible air leaks. Literature recommends that when inflating the ETT cuff, 10 mL of air should be used. However, when more than 10 mL is required, notifying the physician regarding the leak is imperative as the cuff might be damaged, thus requiring re-intubation of the critically ill patient (Saeed et al. 2020).

Thirdly, less than half of the participants in the post-test in both intervention groups indicated that the aspiration of gastric contents and increased chances of ventilator-associated pneumonia are all complications of under-inflation of the ETT cuff, although no statistically significant difference was seen between groups. An ETT cuff pressure below 20 cmH_2_O is regarded as a contributing risk factor for ventilator-associated pneumonia and ineffective positive pressure ventilation (Sadasivan, George & Krishnakumar 2018).

An improvement in knowledge was observed for certain practices but differed per group. For example, both intervention groups showed the most knowledge improvement regarding the complications of under-inflation of the ETT cuff pressure and the frequency of monitoring ETT cuff pressure. However, knowledge only improved significantly in Intervention 2 group, where multiple passive and active implementation strategies were employed, including a half-day educational session, printed materials (passive implementation strategies) and monitoring visits (active implementation strategy). Active approaches, including monitoring or site visits, have been proven particularly effective in the implementation of guidelines but should preferably be part of a multifaceted approach to effectively improve knowledge amongst practitioners (Thomas & Kunzmann 2014).

Age and experience have been associated with the level of knowledge, as older practitioners have often acquired more experience, which usually translates into better knowledge outcomes (Jansson et al. 2013; Thomas & Kunzmann 2014). It could be argued that nurses in the current study scored generally lower in their knowledge related to nursing care practices for the management of ETT cuff pressure because they were relatively young and had less experience than the older, more experienced nurses. Mentoring of the younger and/or more inexperienced nurses by ‘buddying’ them with older, more experienced nurses in the ICU during shifts could assist in increasing knowledge. Mentoring has proven to have positive outcomes on patient care as well as improved job satisfaction amongst nurses in ICUs, which consequently leads to a reduced attrition rate amongst nurses (Kostrey Horner 2017; Sibiya, Ngxongo & Beepat 2018; Vergara 2017). A formal mentoring programme is, therefore, recommended for the ICUs in this study.

In this study, only 21% of the participants indicated that they had undergone formal ICU training. Although it seems to be a common practice for nurses working in ICUs in Malawi and many other lower- and middle-income countries to be trained as they work in the units (Gundo 2019), our study showed that such in-service training may not be adequate to improve nursing care practices, specifically with regard to the management of ETT cuff pressure. Hence, this could explain the generally low knowledge scores obtained for questions regarding nursing care practices for the management of ETT cuff pressure by the nurses in this study. Quality training is imperative for good quality care in the ICU as training is considered as being related to an increase in knowledge regarding ICU-related nursing practices (Haniffa et al. 2017; Perrie et al. 2014). Therefore, it is recommended that nurses in ICUs should be formally trained before positioning them in these units and/or that a structured training programme should be provided to those currently without formal training but working in ICUs.

Furthermore, it remains unclear whether the improvement in knowledge of some practices was internalised by the nurses who participated in the study and whether it would be retained. Continuous professional development, which is critically reflective, constructive, networked and supported with adequate material and technical resources according to nurses’ needs and the specific ICU context, should be encouraged (Price & Reichert 2017). For example, frequent in-service training on all practices related to the management of ETT cuff pressure should be conducted. This is especially important as the overall knowledge score in this study was low; and for some practices, the results showed little improvement in knowledge or even a decline compared to the pre-test questionnaire.

Finally, in Malawi, the non-availability of guidelines for the management of ETT cuff pressure in ICUs could have contributed to inadequate knowledge in this area. This explains the reason for some non-recommended practices regarding the management of ETT cuff pressure, as these guidelines are tools used to standardise treatment plans and assist healthcare providers in making evidence-informed clinical decisions (Jun et al. 2016).

### Limitations of the study

Several limitations were identified in the way this study was conducted. Firstly, it did not include the context of implementation, such as environmental readiness and the stakeholders involved that, according to Harvey and Kitson (2015), should be considered during implementation. Furthermore, this research did not consider the possible barriers and facilitators, which should be assessed in order to tailor the implementation strategies to the specific setting and target group (Fischer et al. 2016). Additionally, the sample size per pre- and post-group for the two groups was too small to conduct inferential statistics for the demographic variables meaningfully and made it impossible to include further variables in the analysis. For this reason, binary coding was the only feasible coding scheme as responses were regarded as either correct or incorrect. Furthermore, we had separate data sets for the pre- and post-tests, and the repeated-measures technique could not be used as it requires a pre- and post-score for each respondent. Finally, because of the anonymity of responses, test–retest correlation which requires pairing respondents’ pre- and post-scores was not possible.

The demographic differences between the pre- and post-tests within both groups were because of the different samples for these groups. However, the participants within each intervention group were the same for the pre- and post-test questionnaires. Randomised sampling could have been used to avoid this but was not possible because of the already small sample. An attempt was made to include all the participants (*N* = 61) in the study, but participation was voluntary; thus, a bigger sample could not be achieved. Furthermore, the participants’ pre- and post-test data could not be matched as participants responded anonymously.

The poor Cronbach’s alphas that were observed for the measurement instrument of this study are something that must be accepted as a limitation of the study, which can be addressed in the follow-up studies with bigger samples as well as a redesign of the measurement instrument, for example, measuring the different facets of the knowledge being investigated, instead of the current single score for all facets combined.

### Recommendations for future research

Finally, although the management of ETT cuff pressure is mainly conducted by nurses, it forms part of a multidisciplinary team approach. For continuity of care, the perspectives of stakeholder other than nurses, such as medical specialists, could have been included in the study. Additionally, examining the educational intervention’s impact in terms of the effect of the improved knowledge regarding managing ETT cuff pressure on patient outcomes should also have been considered. A follow-up study of implementing guidelines using a variety of implementation strategies, testing a larger population and taking into consideration the contextual, demographic and stakeholder issues mentioned would be helpful in such a complex context.

## Conclusion

The results of this quasi-experimental pre- and post-test study showed varied responses amongst nurses regarding their knowledge of nursing care practices in the management of ETT cuff pressure between both intervention groups. Although most nurses were not formally ICU trained, the educational intervention led to a general improvement in their knowledge of nursing care practices for the management of ETT cuff pressure. This was specifically the case for Intervention 2 group where a variety of implementation strategies, including the half-day educational session, printed materials (passive strategies) and monitoring visits (active strategy), significantly improved their knowledge. The implementation of a formal training programme and mentorship programme for nurses working in the ICU in Malawi would be of great benefit to empower nurses with adequate knowledge, skills and appropriate attitudes for the management of the ETT cuff pressure. Follow-up studies would also assist in understanding how guidelines could be implemented effectively to achieve better knowledge outcomes amongst nurses concerning nursing care practices as well as patient outcomes in the ICU context.
